# Evaluation of efficacy and safety of propofol in the treatment of procedural sedation/anesthesia in neonates: A protocol for systematic review and meta-analysis

**DOI:** 10.1097/MD.0000000000027147

**Published:** 2021-09-17

**Authors:** Pei-Xia Yu, Li-Jun Bo

**Affiliations:** a Department of Anesthesiology, the Third Hospital of Hebei Medical University, Shijiazhuang, Hebei, China; b The Second Hospital of Hebei Medical University, Shijiazhuang, Hebei, China.

**Keywords:** anesthesia, efficiency, neonates, procedural sedation, propofol, safety

## Abstract

**Background:**

In newborns, propofol anesthesia is commonly utilized. Propofol is increasingly being shown to be effective and safe in treating procedural sedation and anesthesia in neonates. This research aims to evaluate the efficacy and safety of propofol in neonates using systematic review and meta-analysis methodologies.

**Methods:**

A thorough review and meta-analysis of studies on propofol anesthesia in neonates will be conducted. Conduct comprehensive searches in Web of Science, PubMed, Cochrane Library, EMBASE database, WanFang database, and Chinese biomedical literature database before May 25, 2021, to obtain published and qualified research. Two reviewers will assess the quality of the included papers and extract the data independently. Then, for meta-analysis, we will utilize RevMan 5.3 software.

**Results:**

This study will pool the data of separate trials to analyze the efficacy and safety of propofol in the treatment of procedural sedation/anesthesia in neonates.

**Conclusion:**

Our findings will give strong data for determining whether propofol is an effective treatment for procedural anesthesia in neonates.

## 1 Introduction

Propofol is a common general anesthetic medication used in newborns. It has a number of qualities that make it appealing to neonates. Propofol has a fast beginning of the action, and its therapeutic effect is almost instantaneous after injection (“one arm-brain circulatory time”); it normally induces hypnosis within 20 to 40 seconds of injection.^[1]^ It has an ultrashort half-life and extremely short recovery times following sedation, typically between 5 and 10 minutes, with a peak impact of 92 seconds.^[2]^ Emesis is an uncommon side effect of propofol use. Neonates admitted to neonatal intensive care units or neonates admitted for elective operations frequently undergo elective or semi-elective operations requiring sedation, analgesia, or anesthesia. Procedures on newborns can be stressful and traumatic at times. Side effects are possible with medications used to relieve stress or discomfort in newborns. Propofol is a regularly used sedative in both adults and children for simple procedures and big operations. The lack of a direct analgesic effect, the lack of a reversal agent, respiratory and hemodynamic depression, and a short therapeutic window are all downsides of propofol. Numerous researches have not confirmed the efficacy and safety in the neonatal intensive care units, so there is still a controversy.

## 2 Objectives

This research will undertake subgroup analysis based on propofol administration technique, active control agent type, procedure type, and gestational age. To see if propofol administration in newborns undergoing sedation or anesthesia for procedures is effective and safe.

## 3 Methods and design

The Preferred Reporting Items of the Systematic Review and Meta-analysis protocol will be used to produce this systematic review and meta-analysis.^[3]^ Part of the criteria for contemplating research will be recorded in this protocol, including inclusion and exclusion criteria, study population, type of experiment, outcome measures data, and type of studies.

### 3.1 Eligibility criteria

1. Inclusion criteria for literature
  (1) Randomized controlled or quasi-randomized trials of propofol against placebo/no therapy or another anesthetic regimen for neonates must be included in the literature.
  (2) We will include full-term and pre-mature infants who received any diagnostic and treatment procedures that needed sedation/analgesia/anesthesia (maximum age after delivery was 40 weeks and 28 days after normalizing for gestational age).
  (3) We will compare propofol to placebo, no therapy, or any other sedation/anesthesia regimen.
  (4) We will look into both bolus and continuous propofol infusions, as well as any dose and duration of propofol.
  (5) Chinese and English will be the only languages available.
2. Literature exclusion criteria
  (1) The cross-over studies are not included since there would not be enough time for a “wash out” period, and the effects of specific agents could not be determined.
  (2) The literatures with evident statistical analysis flaws were eliminated.
  (3) Failure to give adequate original data resulting in unproductive data collection.
  (4) Experimentations on animals

### 3.2 Types of outcome measures

Meet the conditions for appropriate sedation/anesthesia:

1. Accomplish the objectives (sedation/anesthesia) without the use of further medications.
2. Achieve adequate sedation/anesthesia (as reported by the trial author).
3. The pressure on the neonates during the operation.
4. Time to complete the procedure.
5. The preparation time of anesthetics.
6. Time to achieve sleep/muscle relaxation.
7. Time to return to the previous clinical state.
8. The safety of propofol (these side effects occur during drug withdrawal or within 24 hours of drug withdrawal).

### 3.3 Literature retrieval

Two reviewers will undertake an independent search for published material. They will search the databases PubMed, EMBASE, the Cochrane Library, the WanFang database, and the Chinese biomedical literature database. The search will be limited to human research, with articles only available in English and Chinese. The most recent literature search was completed on May 25, 2021.

### 3.4 Risk bias and quality assessment

The listed studies’ risk of bias will be evaluated. Two reviewers will screen the literature separately, evaluate the methodological quality, extract the data, and cross-check it. In addition, we will assess the methodological quality of each trial. The information will be included in the characteristics table of the studies included. We will pay close attention to any significant issues linked to other potential sources of bias in each included study. We also intend to assess whether each study is devoid of other concerns that could lead to bias.

### 3.5 Statistical analysis

For this systemic review and meta-analysis, we will use RevMan 5.3 software. When suitable, the plan's statistical parameters are relative risk, risk difference, number needed to treat to acquire additional beneficial benefits, number needed to treat additional harmful results, and mean difference. For estimating treatment effects, we will offer the 95% confidence interval.

## 4 Discussion

Propofol is an intravenous anesthetic that acts quickly and is insoluble in water. It is frequently dissolved in a fat emulsion of long-chain triglycerides. Because of its rapid onset, consistent induction, short duration, rapid and complete awakening, and low incidence of coughing and hiccups. In recent years, propofol has become increasingly popular for inducing general anesthesia during cesarean section both at home and abroad.^[4,5]^ Propofol medium/long-chain fat emulsion injection (medium-chain triglycerides/long-chain triglycerides) contains excipients that are 50% medium-chain triacylglycerol and 50% long-chain triacylglycerol. Experiments reveal that injection of propofol medium/long-chain fat emulsion is an efficient intravenous anesthetic.^[6]^ Propofol can enter the fetus through the placenta due to its high fat solubility. Propofol enters the fetus through the umbilical vein from the placenta and is gradually metabolized in the liver. The remainder is absorbed into the systemic circulation via the inferior vena cava via the venous catheter. The blood-brain barrier is highly permeable, allowing medications to flow easily, yet the concentration of the drug reaching brain tissue is already fairly low. When the fetus is delivered, the blood concentration of the fetus is predicted to be low, which will not severely impair the infant. When used for cesarean delivery, propofol provides numerous advantages. Its metabolism is fast, the blood concentration declines quickly, and the patient wakes up without creating a long-term depression in the infant. The most generally used procedures for predicting and assessing the state of the fetus or infant include fetal heart rate monitoring, umbilical cord blood gas analysis, and the Apgar score. The world perinatal medicine community regards cord blood gas analysis as the most reliable sign for assessing fetal oxygenation and acid-base status, as well as a valuable objective signal for identifying fetal asphyxia.^[7]^ The Apgar score measures the clinical condition of babies following birth. A clinical state index is used to assess neonates’ clinical status following birth, such as breathing, circulation, physiological reflexes, and muscle tension. The later state is associated with the earlier treatment. There is currently no unambiguous high-quality evidence suggesting that propofol should not be administered for general anesthesia during cesarean delivery.^[8]^ Propofol can be used as an anesthesia inducer in routine general anesthesia procedures.^[9,10]^ However, propofol is still used with caution, and the rate and amount of administration should be monitored.

## Author contributions

**Conceptualization:** Pei-Xia Yu, Li-Jun Bo.

**Data curation:** Pei-Xia Yu, Li-Jun Bo.

**Formal analysis:** Li-Jun Bo.

**Funding acquisition:** Li-Jun Bo.

**Investigation:** Pei-Xia Yu, Li-Jun Bo.

**Project administration:** Li-Jun Bo.

**Resources:** Pei-Xia Yu, Li-Jun Bo.

**Software:** Pei-Xia Yu.

**Supervision:** Pei-Xia Yu, Li-Jun Bo.

**Validation:** Pei-Xia Yu.

**Visualization:** Li-Jun Bo.

**Writing – original draft:** Pei-Xia Yu, Li-Jun Bo.

**Writing – review & editing:** Pei-Xia Yu, Li-Jun Bo.
